# Impact of climate change on waterborne infections and intoxications

**DOI:** 10.25646/11402

**Published:** 2023-06-01

**Authors:** Susann Dupke, Udo Buchholz, Jutta Fastner, Christina Förster, Christina Frank, Astrid Lewin, Volker Rickerts, Hans-Christoph Selinka

**Affiliations:** 1 Robert Koch Institute Centre for Biological Threats and Special Pathogens Berlin, Germany; 2 Robert Koch Institute Department of Infectious Disease Epidemiology Berlin, Germany; 3 German Environment Agency Department of Drinking Water and Swimming Pool Water Hygiene Berlin, Germany; 4 German Environment Agency Department of Drinking Water and Swimming Pool Water Hygiene Bad Elster, Germany; 5 Robert Koch Institute Department of Infectious Diseases Berlin, Germany; 6 German Environment Agency Department of Environmental Hygiene Berlin, Germany

**Keywords:** NON-CHOLERA VIBRIO, LEGIONELLOSIS, CYANOBACTERIA, ENVIRONMENTAL MICROBES, VIRAL INFECTIONS

## Abstract

Progressive climate change holds the potential for increasing human health risks from waterborne infections and intoxications, e. g. through an increase in pathogen concentrations in water bodies, through the establishment of new pathogens or through possible changes in pathogen properties. This paper presents some examples of potential impacts of climate change in Germany. Non-cholera *Vibrio* occur naturally in seawater, but can proliferate significantly in shallow water at elevated temperatures. In the case *of Legionella*, climate change could lead to temporary or longer-term increased incidences of legionellosis due to the combination of warm and wet weather. Higher temperatures in piped cold water or lower temperatures in piped hot water may also create conditions conducive to higher *Legionella* concentrations. In nutrient-rich water bodies, increased concentrations of toxigenic cyanobacteria may occur as temperatures rise. Heavy rainfall following storms or prolonged periods of heat and drought can lead to increased levels of human pathogenic viruses being washed into water bodies. Rising temperatures also pose a potential threat to human health through pathogens causing mycoses and facultatively pathogenic micro-organisms: increased infection rates with non-tuberculous mycobacteria or fungi have been documented after extreme weather events.

## 1. Introduction

Numerous human pathogens can be transmitted through contact with water. Oral ingestion, inhalation or skin contact with pathogens such as *Legionella*, *Vibrio*, cyanobacteria or non-tuberculous mycobacteria lead to diseases, which can be severe and run fulminant courses. Pathogens causing mycoses and other facultatively pathogenic micro-organisms also present a health hazard.

The consequences of global warming and resulting climate change are expected to have effects on waterborne infections in the future. Longer seasonal warm periods due to hot summers or prolonged warm autumns can lead to an increase in water temperatures in northern latitudes. These increased temperatures can favour the multiplication of pathogens. Such a climatic effect could be shown, for example, for the Baltic Sea by means of satellite data since 1990. An analysis showed that the annual mean temperature of the surface of the entire Baltic Sea has warmed by around 0.8°C over the last 16 years [1]. Storm surges and floods can also spread waterborne germs, especially waterborne viruses and facultatively pathogenic environmental microbes. It can be assumed that these weather phenomena will occur more frequently as climate change progresses.

Many waterborne infections can be acquired during leisure activities. This has far-reaching consequences for human health, as the leisure behaviour of the population may become more oriented towards water-associated activities in the context of rising outdoor temperatures. In this article, the risk of waterborne infections and intoxications in Germany will be discussed using the example of various micro-organisms whose occurrence is influenced by climate change.

The contents to be presented in this article were jointly determined by the authors according to their expert opinions. Appropriate references to current literature have been added, but there is no claim to completeness.

## 2. Non-cholera *Vibrio*

*Vibrio* are gram-negative, rod-shaped bacteria that are moderately to markedly halophilic (salt-loving). *Vibrio (V.) cholerae* O1/O139, which can produce the cholera toxin, are probably the best-known representatives of *Vibrio*. They cause epidemic cholera. Cholera is occasionally diagnosed in Germany as a travel-associated infection, but is not endemic in Europe.

However, so-called non-cholera *Vibrio* (NCV) such as *V. parahaemolyticus*, *V. vulnificus*, *V. cholerae* non-O1/non-O139, *V. fluvialis*, *V. furnissii*, *V. alginolyticus*, *V. mimicus* and *V. metschnikovii*, which are also pathogenic to humans, occur as components of the normal bacterial flora in the North Sea and Baltic Sea, and occasionally in somewhat salinic inland waters. The North and Baltic Seas contain slightly different compositions of NCV, presumably due to differences in salinity. In a study investigating water and sediment, it was shown that in the highly saline North Sea, *V. parahaemolyticus* is found more frequently, whereas in the Baltic Sea mainly *V. vulnificus* is detected besides other NCV. *V. cholerae* non-O1/ non-O139 is also frequently detectable here [2]. Shallow waters poorly mixed by wind, tides or other currents are particularly affected, as they can heat up strongly when exposed to sunlight. At water temperatures above 20°C, NCV can proliferate in these waters. On the North Sea coast, estuary beaches are particularly susceptible to the occurrence of higher NCV concentrations due to the reduced salinity. On the Baltic Sea coast, ‘bodden’ waters (briny coastal lagoons in the shelter of islands) in summer and early autumn fulfil many conditions for NCV growth. Predictions of NCV proliferation can be seen in the 'Vibrio map viewer', a tool of the European Centre for Disease Control (ECDC) that uses real-time data on water temperature and sea surface salinity to predict the occurrence of environmental conditions that favour the proliferation of *Vibrio*. [3].

People can become infected with NCV in various ways: they may ingest the pathogens in raw or inadequately heated food of marine origin, such as oysters or other seafood [4]. Another article in this status report discusses climate change impacts on foodborne infections and intoxications in more detail (Dietrich et al. [5]). Pathogens can enter the body when larger wounds, or even very small skin lesions, come into contact with water. In this context, infections with *V. vulnificus* are frequently described. Infections through skin injuries occurring directly in the water are rare, yet possible [6, 7] and so are wounds incurred when handling animals, shells and stones to which seawater adheres, for example when processing fish. Especially in children, ear infections with NCV, mainly with *V. cholerae* non-O1/non-O139, occur frequently, e. g. through swimming or bathing in shallow waters.

Depending on the various routes of infection and pathogen species, different clinical pictures manifest in the form of gastroenteritis, wound infections or ear infections; in the case of aspiration of seawater, even pneumonia, caused by *V. vulnificus*, has been described [8, 9]. Gastroenteritis usually does not correspond to extremely severe, rice water-like diarrhoea with a great risk of dehydration typical for the clinical picture of cholera, since NCV lack the cholera toxin. Starting from wounds, or injuries to the skin barrier, the bacterial toxins can cause invasive, cross-tissue, usually purulent infections, requiring urgent surgical treatment [10]. Both wound infections and gastroenteritis can lead to sepsis, which is associated with significant lethality. Rapid medical treatment is necessary. While infections with NCV can affect all age groups, clear risk groups can be identified, particularly in the case of severe wound and soft tissue infections and sepsis: these include older and immunocompromised persons. People with pre-existing conditions such as diabetes mellitus, liver disease (e. g. liver cirrhosis, chronic hepatitis), cancer (e. g. after chemotherapy) and severe heart disease also have an increased risk of symptomatic infection and of a severe course of disease [11]. In contrast, young healthy adults are rarely among the cases recognised in Europe, and they usually do not become severely ill. With rapid, appropriate and adequately dosed antimicrobial therapy, infections can be controlled even in high-risk patients. If left untreated or treated too late, surgical intervention (up to and including amputation of affected limbs) may also be necessary due to the rapid progression of the infection. In case of injuries or visible infections of skin and soft tissues post salt water exposure, a current guideline on skin and soft tissue infections therefore recommends the immediate application of antibiotic combination therapy [12]. Therapy should be started in patients at risk even while the microbiological confirmation of NCV is pending [9]. Patients with gastroenteritis caused by NCV who are at increased risk of sepsis due to pre-existing conditions such as diabetes or liver damage should also receive antibiotic therapy at an early stage.

In the years prior to the introduction of an explicit mandatory notification requirement, up to 20 cases of NCV infections with exposure sites in Germany were reported to the Robert Koch Institute (RKI, Germany’s national public health institute) each year. These cases were diagnosed more frequently in the warmer summers of the years 2003, 2006, 2010, 2018 and 2019.

A large study of 63 German cases in the years 2018 and 2019, which had very hot summers, described patients with a clear age and gender distribution (the majority was over 60 years old and predominantly male) as well as seasonality of the infections. Patients had become infected with NCV significantly more often at or in the Baltic Sea than at or in the North Sea. Wound infections were the most frequent form of disease, with the majority (84%) being pre-existing wounds inflamed by seawater contact. Common pre-existing conditions of the mostly older persons were cardiovascular disease, diabetes mellitus or immunodeficiency; 51% of the patients had to be treated in an intensive care unit, and a total of eight patients died in connection with the infection [9].

On 01.03.2020, in addition to a mandatory notification requirement for cholera in Germany, a mandatory notification requirement for infections with other human pathogenic *Vibrio* was included in the German Protection against Infection Act (Infektionsschutzgesetz, IfSG). A subset of the NCV infections reported since 2020 is described in Table 1. A clear seasonality of disease onset in the summer months among the mainly older patients becomes apparent. It is also evident that the majority of infections with NCV are associated with water contact (especially at the Baltic Sea). With advancing climate change an increase in these infections, especially in coastal waters, is to be expected during hot, long summers.

### 2.1 Influence of climate change on *Vibrio* infections

Climate change affects infections with *Vibrio* in at least two ways:

Since water temperatures above 12°C generally have a favourable effect on the occurrence of *Vibrio*, and NCV can multiply particularly strongly in warm water above 20°C, more frequent longer warm periods contribute to the concentration of pathogens in the water. This concentration can be additionally increased if the water circulation is reduced or even absent due to altered tidal fluctuations, lower frequency of storms or changed influences of other currents. A climate-induced extension of that period of the year in which high NCV concentrations are to be expected also extends the phase in which vulnerable people in particular can come into contact with the pathogens, e. g. by extending the bathing season. In addition, it should be noted that demographic change is generally increasing the proportion of vulnerable groups in the German population, and presumably also among holidaymakers on German coasts. As a consequence of global warming, it is expected that the surface temperature in the Baltic Sea will increase by about 3°C to 4°C in the next decades [13], which will lead to a general increase of NCV pathogenic to humans in coastal waters.

### 2.2 Recommendations to limit exposure to NCV

Due to the breadth of this topic, the recommendations presented here focus on wound infections and sepsis, primarily caused by *V. vulnificus*, as these are the most serious NCV infections in Germany. Studies concerning *V. vulnificus* as a human pathogen have already been described in detail, and show a significantly increased risk of certain groups of people becoming infected with this pathogen and becoming seriously ill [14].

(1) Infections with NCV can be reduced if potentially infectious water contact is avoided, in particular, wounds should not be exposed to seawater. Theoretically, it is helpful to opt for water contact with lower infection probabilities, such as wading or swimming at beach sections open to the sea and influenced by tides, instead of particularly poorly mixed and brackish coastal sections such as ‘bodden’ waters. People who bear the highest risk of a severe course of the disease in the event of infection with NCV would benefit most from these measures, above all senior citizens, especially those with the described pre-existing conditions, e. g. poorly healing wounds on the legs. Severe courses of the disease can be prevented or mitigated by rapid and appropriate treatment.

(2) It is a widespread piece of misinformation that seawater disinfects wounds. Patients with risk factors for severe disease progression should be informed, e. g. by their general practitioners, about the general risks of infection when wounds come into contact with natural waters. Coastal rehabilitation clinics and similar facilities should seasonally actively inform residents about the risks of infection with NCV and provide advice on how to avoid infection. Tourist facilities should also address the relevant risks in a manner appropriate to the target group. Physicians, especially in coastal areas, should consider NCV infections as a differential diagnosis, particularly in the case of wound infections and sepsis requiring immediate treatment after coastal exposures, and initiate treatment with suitable antibiotics as quickly as possible.

## 3. *Legionella*

Legionnaires' disease (LD) is a form of pneumonia caused by *Legionella*, largely the species *Legionella pneumophila.* Typically, this bacterium is found in water systems or biofilms, but it must be nebulised and then inhaled to cause illness [15]. Epidemiologically, three categories of LD are distinguished: travel-associated, hospital-associated, and community-acquired, i. e. acquired in a personal or work environment. In principle, transmission of *Legionella* is possible through a variety of sources, e. g. aerosols from evaporative cooling systems [16, 17] or hot tubs [18], but according to current knowledge, the epidemiologically most important source of infection is domestic drinking water [19]. However, in studies searching for sources of infection with LD, at least half of the sporadic illnesses (i. e. not occurring in the context of an outbreak) remain without a confirmed source of infection [19, 20]. A proportion of these could occur – including via alternative routes of transmission – in the context of certain weather conditions (e. g. increased rainfall) [21]. Major outbreaks of LD are relatively rare in Germany and have often been caused by evaporative cooling systems in the past, e. g. 2009/2010 in Ulm [22] or 2013 in Warstein [23]. LD is a seasonal disease, with most cases occurring in the summer and autumn months. Of the three epidemiological groups, seasonality is most pronounced in travel-associated cases, followed by community-acquired cases. Hospital-associated cases, on the other hand, show little seasonality.

In Germany, LD is notifiable according to IfSG. The annual incidence is about 1.9:100,000 inhabitants, corresponding to about 1,500 reported cases per year. However, an unreported number of 15,000 to 30,000 cases is assumed [24]. Community-acquired cases of LD are the most common, accounting for about 70%, followed by travel-associated cases (about 20%) and cases associated with a stay in a hospital or nursing home (about 10%). Older persons, especially males, and people with pre-existing heart, lung or other organ disease are considered to be at risk. Smoking is a strong risk factor [25].

### 3.1 Influence of climate change on legionellosis

Climate change could affect the frequency of LD occurrence in two ways:

(1) Through environmental factors, which can sometimes mimic outbreaks: increased incidence of LD was associated with various weather conditions in some studies. These included increased humidity [26–29], increased precipitation [26–31], increased air temperature [26, 29, 31], low air pressure [30], or combinations of these factors. In the Netherlands, outbreak-like increases in incidence have been associated with warm, humid weather conditions [26]. In Germany, there were unexpected increases in case numbers in the summer of 2018 in Bavaria and Baden-Württemberg, which were probably associated with a similar effect. The mechanism of the environmental factor-associated incidence increase is unknown. Some authors have hypothesised that driving cars on wet roads may lead to aerosolisation of puddle water contaminated with *Legionella* [28, 32]. This could explain the fact that in a Japanese study, *Legionella* DNA was identified in air samples near busy roads, sometimes even *L. pneumophila* [28]. The amount of *Legionella* DNA correlated with the monthly amount of precipitation. In places where climate change leads to a more frequent coincidence of warm and humid weather, it cannot be ruled out that the incidence of LD may increase and that occasional outbreak-like clusters of cases may occur.

(2) In the long term, through household drinking water: given the rising average air and ground temperatures, it is possible that the base temperature of cold water will increase. Such an effect could lead to increased *Legionella* growth in cold water, which in turn could lead to increased *Legionella* concentrations in both cold and hot water. Another factor that could lead to increased *Legionella* growth is the increasing desire of many households to save energy by lowering hot water temperatures, both to protect the climate and to save money as energy prices rise. This could push the incoming hot water temperature into a range, say below 50°C, where *Legionella* growth is not only not suppressed but may even be encouraged. It is reasonable to assume an increased risk in the context of climate change if one accepts the premise that a higher concentration of *Legionella* in drinking water is associated with an increased risk of LD. However, the notion of infective dose paradox has existed for a long time [33], i. e. it has been observed that a higher *Legionella* concentration is not necessarily associated with an increased risk of LD. In a case-control study conducted in Berlin (the LeTriWa study), the strongest risk factor identified at the microbiological level was not *Legionella* concentration, but the presence of virulence-associated (MAb 3/1-positive) *Legionella* in domestic drinking water [19].

### 3.2 Recommendations to limit the health impact of *Legionella*

There are a number of options for the prevention of LD. In the context of current climate warming, it is important to develop research approaches to further investigate the possible mechanisms, mentioned above, by which climate change influences the incidence of LD.

(1) On the technical side, there is still a considerable need for research. For example, it has been shown that even in the 2011 version of the German Ordinance on the Quality of Water Intended for Human Consumption (Trinkwasserverordnung, TrinkwV), drinking water installations that are not subject to mandatory testing (especially apartments with decentralised drinking water heating, e. g. instantaneous water heaters) may well contain high concentrations of *Legionella* [34]. Furthermore, the LeTriWa study found that virulence-associated *Legionella* were identified not only in drinking water installations subject to mandatory testing, but also in drinking water installations not subject to mandatory testing, and could be associated with the occurrence of LD [19, 34–36]. The influence of water temperature in drinking water installations, *Legionella* concentration and strain type on the risk of LD needs to be investigated. New or modified prevention options could result from the findings, and their effectiveness should be tested.

(2) It is also important to determine which preventive factors or behaviours that can be influenced by individuals help to prevent the transmission of LD. In the LeTriWa study, it was found that personal knowledge of *Legionella* and its characteristics as well as individual, preventive behaviours can reduce the risk (Buchholz et al.; data not published). One behaviour identified as significant was letting water run before use.

(3) Since the negative and very strong effect of smoking as a risk factor for the occurrence of LD is well established, campaigns to reduce smoking against this background may be effective. Further research should investigate how the co-benefits of reduced tobacco consumption can be better communicated.

## 4. Cyanobacteria

Cyanobacteria are gram-negative bacteria, but differ from other bacteria in their ability to perform oxigenic photosynthesis. Because of their ecology, which is similar to that of algae, and the possession of accessory pigments such as the blue phycocyanin, cyanobacteria are sometimes called ‘blue-green algae’. They colonise various habitats worldwide and are a natural part of the biotic community in water bodies. However, in waters with high nutrient concentrations (phosphorus, nitrogen), cyanobacteria can proliferate massively, with negative effects on the ecosystem and on the use of these waters as a drinking water resource or bathing water, especially because of the ability of some cyanobacteria to produce potent toxins [37].

Especially those cyanobacteria commonly found in freshwater such as *Microcystis*, *Planktothrix, Aphanizomenon*, and *Dolichospermum* are potentially toxic and form large populations, so-called algal blooms, under favourable conditions. Not all genotypes of cyanobacteria can produce toxins, and populations usually consist of a mixture of non-toxic and toxic genotypes, the latter with sometimes marked differences in toxin content. Toxin concentration in a water body depends largely on the biomass of cyanobacteria, but also on the genotype composition. Environmental factors influence both the occurrence and extent of cyanobacterial populations as well as genotype composition, which can thus change over the course of the season.

Cyanobacteria can be harmful due to their ability to produce toxins, but do not multiply in the human body. The most important cyanobacterial toxin groups include hepatotoxins (microcystins, nodularins, cylindrospermopsins) and neurotoxins (anatoxins, saxitoxins) [37]. The systemic effect of the toxins occurs exclusively by oral uptake; uptake via the skin is not likely. For hepatotoxins, chronic toxicity is of primary importance, whereas the health risk for neurotoxins is primarily due to their acute oral toxicity, which may be pronounced.

Allergic effects of cyanotoxins have not been demonstrated so far, but irritating and sensitising properties have been observed in skin contact with unnaturally high concentrations of cylindrospermopsin. The symptoms often reported in connection with cyanobacterial contact, such as mucosal irritation, nausea, and respiratory illness, are most likely not caused by cyanotoxins, but are due to other cell components, bacteria accompanying cyanobacteria, or other pathogenic organisms in the water [37]. A clear causality in the case of rather unspecific symptoms is usually not demonstrable due to the large number of possible causes.

Humans may be exposed to cyanobacteria and their cyanotoxins during recreational activities in waters with algal blooms, but also through drinking water if drinking water treatment does not effectively remove cyanotoxins from infested waters. Furthermore, they may be present in food (e. g. fish, food supplements made from cyanobacteria). Clearly documented serious illnesses or even deaths in humans due to cyanotoxin ingestion are only known in isolated cases internationally, and not at all in Germany.

### 4.1 Influence of climate change on cyanobacteria

The primary cause of mass occurrence of cyanobacteria is increased nutrient concentrations (phosphorus, nitrogen) in the water body. The effects of climate change can increase or decrease the occurrence of cyanobacteria. In nutrient-rich waters, for example, stable stratification caused by high temperatures has a favourable effect on growth. In contrast, in water bodies with lower nutrient levels, falling water levels due to low precipitation on the one hand and nutrient run-off from agricultural land during heavy rainfall events on the other hand can lead to an increase in nutrients and thus to more cyanobacteria [38, 39]. However, pronounced stratification of a water body with incomplete mixing can lead to a decrease in nutrients, which in turn can negatively impact cyanobacteria. Heavy rainfall and wind can also have a detrimental effect on their proliferation [40]. Since the data on the influence of environmental factors on the toxin content of a population are not unambiguous, it is not yet possible to assess the overall influence of climate change.

In summary, it is very likely that climate change will lead to significant changes in ecological processes in water bodies. It is currently difficult to estimate how these changes will affect individual water bodies: in some waters this will lead to more frequent and more pronounced cyanobacterial blooms, but in others it will not. As this depends on the trophic state and morphometry of a water body as well as regional weather phenomena, no general influence of climate change on cyanobacterial blooms can be derived for all water bodies [39].

### 4.2 Recommendations to limit the health impact of cyanobacteria

For the most important toxins, the World Health Organization (WHO) has set guideline values for both drinking water and bathing water [37]. The EU Drinking Water Directive, revised in 2021, adopts the WHO guideline value of 1 μg/l for microcystin-LR; this is to be transposed into national law by 2023. For bathing waters, article 8, ‘Cyanobacterial risks’, of the EU Bathing Water Directive regulates the risk assessment and communication concerning toxic cyanobacteria.

For drinking water, no risk from cyanotoxins is to be expected due to the low proportion of treated surface water in Germany and effective drinking water treatment processes in accordance with legal requirements. On the other hand, bathing in waters heavily infested with cyanobacteria may pose a health risk, as most cyanobacterial blooms in Germany contain cyanotoxins. Especially the ingestion of larger quantities of contaminated water must be avoided, which is why children playing in the surf (due to their frequent hand-mouth contact), children learning to swim, or people engaging in water sports exposed to aerosols (e. g. when water skiing) belong to the risk group. However, the largest amounts of water are probably ingested in bathing accidents (near-drowning).

In order to protect bathers, bathing waters reported to the EU are examined for the presence of cyanobacteria and, depending on the extent of contamination, warnings or even a bathing ban are issued [41]. In addition, education is necessary for responsible action by individuals, since water bodies cannot always be examined promptly and dense algal blooms close to the shore can occur sporadically.

Finally, the most sustainable protection against cyanobacteria is to prevent their (mass) proliferation in the water body. This can only be achieved by ensuring sufficiently low concentrations of nutrients and is also imperative in order to control the effects of climate change [42].

## 5. Waterborne viral infections

In contrast to the clearly traceable effect of climate change on the proliferation of vector-borne viruses (see the article in this status report on the effects of climate change on vector-borne infectious diseases, Beermann et al. [43]), the effects of climate change on human pathogenic enteric viruses are not immediately apparent. Gastrointestinal infections caused by human pathogenic enteric viruses, such as noroviruses, rotaviruses, enteroviruses, and hepatitis A and hepatitis E viruses, which can cause waterborne infections through contamination of water bodies, have declined in Europe in recent decades due to hygienic measures. Due to the COVID-19 pandemic and the resulting hygiene measures (reduced contacts, lockdowns and the wearing of face masks), which led to a general reduction in the circulation of human pathogenic viruses, little attention has been paid to changes in the seasonal occurrence and biodiversity of enteric viruses in water bodies, which can be significant.

### 5.1 Influence of climate change on waterborne viral infections

Waterborne infections by enteric viruses are strongly affected by the secondary effects of climate change [44]. Human pathogenic viruses that enter the aquatic environment can often maintain their infectivity for long periods of time, depending on the stability of their viral capsid. However, the viruses can no longer replicate in water. Therefore, an increase in water temperature usually does not play a central role for enteric viruses. However, increased concentrations of the pathogens in water are caused mainly by climate change-induced storms, prolonged periods of heat with drought, and heavy rainfall. Such extreme weather events often lead to increased run-off of pathogenic viruses into water bodies and thus to the deterioration of their hygienic quality [45]. The risk of infection may also be increased during dry periods due to reduced water volume and flow rates in rivers. These temporarily increased viral loads lead to increased risks of transmission of waterborne infections and gastrointestinal diseases. Extreme weather events with heavy rainfall and flooding are also becoming more frequent in Germany, as demonstrated by the floods in Rhineland-Palatinate and North Rhine-Westphalia in July 2021, which led to an increased risk of infections with gastrointestinal pathogens due to mixing of wastewater and floodwater. The influence on health of extreme weather events caused by climate change is considered in more detail in another article in this status report (Butsch et al. [46]).

### 5.2 Recommendations to limit the health impact of waterborne viral infections

(1) The majority of newly discovered or recurrent pathogens are viruses [47]. Due to changes in climatic conditions, the emergence of new zoonotic pathogens and an increase in new waterborne viral infections can also be expected in water. Efficient methods for the detection of pathogens and their elimination must therefore be developed.

(2) Furthermore, the influence of climate change on observed changes in the seasonal occurrence of potentially pathogenic viruses in water bodies needs to be investigated in more detail. Water-based epidemiology, as well as the One Health and Planetary Health concepts, which include a consideration of environmental conditions on infection events, offer good frameworks for this. Especially after extreme weather conditions, increased monitoring programmes on the occurrence of pathogenic viruses in water bodies may detect climate-related increases in waterborne virus infections at an early stage and reduce or prevent them by uncovering possible chains of infection.

## 6. Facultatively pathogenic environmental microbes

Numerous facultatively pathogenic micro-organisms (e. g. fungi, amoebae, bacteria, mycobacteria) are part of polymicrobial communities in various environmental habitats (e. g. in soil and water). Compared to non-pathogenic microbes, they are characterised by thermotolerance, i. e. the ability to grow at human body temperature. In contrast to infections caused by classical bacterial pathogens, the diagnosis, treatment and control of infections caused by such environmental microbes present particular difficulties. Microbiological diagnosis is limited due to often slow growth and ubiquitous occurrence. Many of these pathogens are difficult to treat due to antimicrobial resistance. Longer incubation periods may lead to outbreaks being detected late or not at all without molecular typing.

### 6.1 Influence of climate change on facultatively pathogenic environmental microbes

It has been hypothesised that the increase in environmental temperatures provides a selection advantage for certain facultatively pathogenic environmental microbes. In addition, an adaptation of previously non-pathogenic fungi to higher temperatures has been reported, and as a result, these fungi can be considered infectious agents [48]. The establishment of micro-organisms from warm tropical regions in the temperate climate zones of the North American west coast, observed in an outbreak of the tropical fungal pathogen *Cryptococcus gattii*, demonstrates the dynamics of habitat change [49].

Especially after extreme weather events, humans may be increasingly exposed to these micro-organisms due to such changes in pathogen characteristics and distribution. After floods, for example, events like higher rates of infection with non-tuberculous mycobacteria or fungi are increasingly being documented, supporting this hypothesis (e. g. [49–51]).

### 6.2 Recommendations to limit the health impact of facultatively pathogenic environmental microbes

(1) Comprehensive monitoring of the microbial population is necessary in order to document changes in microbial components of environmental habitats and assess risk, e. g. by metagenomic surveillance of micro-organisms in environmental samples using methods that are also suitable for these pathogen groups [52].

(2) Improving diagnostic methods (e. g. polymerase chain reaction (PCR), sequencing) for these pathogens and polymicrobial infections are crucial for successful therapeutic strategies.

(3) The development of molecular typing schemes is necessary for the detection and containment of outbreaks.

## 7. Conclusion and outlook

Global warming and progressive climate change have far-reaching impacts on human health. Waterborne infections and intoxications pose an increased risk in this context. Pathogens that can cause human infections and intoxications through contact with water may become more prevalent as water temperatures rise. Extreme weather events can also lead to increased exposure to aquatic and terrestrial pathogens. Due to changes in climatic conditions, the emergence of new zoonotic pathogens in water and an increase in new waterborne viral infections can also be expected. Climatic changes may also create selection advantages for thermotolerant facultatively pathogenic microbes, and temperature adaptation of previously nonpathogenic micro-organisms may occur.

In this article, we give recommendations to better protect human health from waterborne infections and intoxications in three broad categories: measures to reduce the risk of exposure, education, and research.

Reducing the risk of exposure is the first priority, especially for infections with NCV, but also for cyanobacteria and cyanotoxins. The most sustainable protection against cyanobacteria is to prevent their (mass) proliferation in water. This can only be achieved by ensuring sufficiently low concentrations of nutrients to protect bathers. To prevent infections with NCV and minimise the risk of severe disease, it is important for persons belonging to risk groups to avoid potentially infectious water contact and to seek prompt treatment with antibiotics if infection is suspected.

Since people with pre-existing conditions, immunocompromised and older persons have an increased risk for waterborne infections, improved education about the risks is of great importance. Medical and tourist facilities at the North Sea and Baltic Sea also need to be informed about the increasing risks, especially regarding NCV infections.

Research efforts on risk factors and their control should be increased, for example regarding tobacco consumption as a risk factor for LD. Efficient methods for the detection of different and novel pathogens and their elimination should be developed. Particularly after extreme weather conditions, wide-ranging studies on the occurrence of pathogenic viruses and other microbes in bodies of water may be able to document potential hazards that can arise and thus enable climate-related increases in waterborne infections to be detected at an early stage.

Joint efforts by many actors are therefore required to reduce the increasing risk to human health from waterborne infections and intoxications that can be expected in the context of climate change.

## Key statement

- People with pre-existing conditions are at increased risk of waterborne infections, including older persons and the immunocompromised.
- An increase in infections by non-cholera *Vibrio*, especially in the coastal waters of the Baltic Sea, is to be expected as climate change progresses.
- Climate change could lead to increases in the incidence of legionellosis.
- Climate change will lead to an increase in (potentially toxic) cyanobacteria in some water bodies.
- Extreme weather events can lead to increased exposure to aquatic and terrestrial pathogens.
- Changes in climatic factors can lead to a selection advantage for thermotolerant facultatively pathogenic microbes as well as to a temperature adaptation of previously non-pathogenic micro-organisms.

## Figures and Tables

**Table 1:** Excerpt of suspected non-travel-associated NCV infections reported to the Robert Koch Institute since the introduction of a mandatory notification requirement for NCV Source: SurvNet, database of infectious diseases with mandatory notification requirement in Germany

	2020 (from March)	2021
Cases not explicitly associated with international travel	13 (out of a total of 13 reported NCV infections)	25 (out of a total of 29 reported NCV infections)
Where infection was acquired (where known)	9 x Baltic Sea 1 x North Sea	14 x Baltic Sea 2 x inland waters
Male gender	9 (69%)	16 (64%)
Age range (in years)	22–87, median: 60	8–89, median: 68
Onset of disease July through September	9 (100% of those with onset indicated)	16 (80% of those with onset indicated)
Pathogen	8 x *V. vulnificus* 4 x *V. cholerae* non-O1/non-O139 1 x *V. parahaemolyticus*	14 x *V. vulnificus* 4 x *V. cholerae* non-O1/non-O139 3 x *V. alginolyticus* 2 x *V. parahaemolyticus* 1 x other species 1 x co-infection with 2 species
Forms of disease (where known)	5 x wound infection/sepsis 2 x ear infection	18 x wound infection/sepsis 3 x gastroenteritis 1 x ear infection

NCV = non-cholera *Vibrio*, V. = *Vibrio*
